# Complexity in speech and music listening via neural manifold flows

**DOI:** 10.1162/netn_a_00422

**Published:** 2025-03-05

**Authors:** Claudio Runfola, Matteo Neri, Daniele Schön, Benjamin Morillon, Agnès Trébuchon, Giovanni Rabuffo, Pierpaolo Sorrentino, Viktor Jirsa

**Affiliations:** Aix-Marseille Université, INSERM, INS, Institut de Neurosciences des Systèmes, Marseille, France; Aix-Marseille Université, CNRS, INT, Institut de Neurosciences de la Timone, Marseille, France

**Keywords:** Neural manifolds, Music perception, Speech perception, Resting state, Neuronal population dynamics, Dimensionality reduction

## Abstract

Understanding the complex neural mechanisms underlying speech and music perception remains a multifaceted challenge. In this study, we investigated neural dynamics using human intracranial recordings. Employing a novel approach based on low-dimensional reduction techniques, the Manifold Density Flow (MDF), we quantified the complexity of brain dynamics during naturalistic speech and music listening and during resting state. Our results reveal higher complexity in patterns of interdependence between different brain regions during speech and music listening compared with rest, suggesting that the cognitive demands of speech and music listening drive the brain dynamics toward states not observed during rest. Moreover, speech listening has more complexity than music, highlighting the nuanced differences in cognitive demands between these two auditory domains. Additionally, we validated the efficacy of the MDF method through experimentation on a toy model and compared its effectiveness in capturing the complexity of brain dynamics induced by cognitive tasks with another established technique in the literature. Overall, our findings provide a new method to quantify the complexity of brain activity by studying its temporal evolution on a low-dimensional manifold, suggesting insights that are invisible to traditional methodologies in the contexts of speech and music perception.

## Author Summary

Understanding how the human brain processes speech and music is a fascinating and complex challenge. Our study explores how brain activity changes when people listen to naturalistic speech compared to when they listen to music or when they are at rest. We found that both speech and music engage the brain in more complex patterns of activity than rest, with speech leading to even greater complexity. To achieve this, we used a novel method to study the brain's activity in a simplified, low-dimensional space, representing the dynamical evolution of brain activity across different regions. This approach highlights the demands placed on brain function during the perception of speech and music, providing new insights into how we process these auditory experiences.

## INTRODUCTION

Music and speech processing are among the most complex, characteristically human, cognitive functions. They rely on the modulation of acoustic parameters to yield abstract representations of sound sequences, have a large range of time constants, and contain significant information. In both, complexity is built up following a hierarchical organization by which a finite set of elements (phonemes or notes) are combined into more complex entities (words and melodies), which, in turn, are organized to generate more complex ensembles (such as sentences or refrains), arranged across several other levels to produce natural speech and music (Krumhansl, 2001; Zatorre, Belin, & Penhune, 2002). How the human brain attributes meaning and qualities to sounds remains an open and intricate question (Birbaumer, Lutzenberger, Rau, Braun, & Mayer-Kress, 1996; Carpentier et al., 2020; Neri et al., 2023; te Rietmolen et al., 2024).

A long tradition in neuroscience supports the hypothesis that the interactions between neural units at different spatiotemporal scales are fundamental for sustaining complex cognitive functions and interpreting such stimuli (Bressler & Menon, 2010; Hebb, 1949). Complexity arising from finely tuned interactions among neuronal populations may be mirrored in low-dimensional patterns of interdependence among neural signals. To study these, a great deal of success was obtained by methodologies and theoretical approaches that allow a low-dimensional representation of multivariate brain recordings (Jirsa, 2020). A growing body of evidence demonstrates that the dynamics of the interactions plays a crucial role in sustaining cognitive tasks (Gonzalez-Castillo et al., 2023; Pillai & Jirsa, 2017; Shine et al., 2019), including speech (Gardner et al., 2022; Ji et al., 2015; Martin et al., 2019; Mercier et al., 2022; Nguyen, Karavas, & Artemiadis, 2018; Orepic et al., 2024) and music (Darcourt, 2023) perception.

However, quantifying and comparing the complexity of the temporal evolution of low-dimensional brain dynamics underlying speech or music tasks (beyond focusing on topological properties and task-related segmentation of the latent space) remains challenging. Furthermore, most previous works employed low-dimensional descriptions of brain activity to investigate simple tasks that allow the isolation of cognitive features of interest in controlled scenarios, but provide limited ecological validity (Gardner et al., 2022; Ji et al., 2015; Martin et al., 2019; Mercier et al., 2022; Nguyen et al., 2018). Moreover, while short and simple stimuli in the case of speech might still induce linguistic processing, they are often not cognitively relevant to music listening (Neri et al., 2023; te Rietmolen et al., 2024; Ding et al., 2017).

Another relevant point concerns brain recording techniques. As an example, functional magnetic resonance imaging (fMRI) fails to capture the fast dynamics of music and speech due to its low temporal resolution (Gardner et al., 2022).

**Figure 1.**
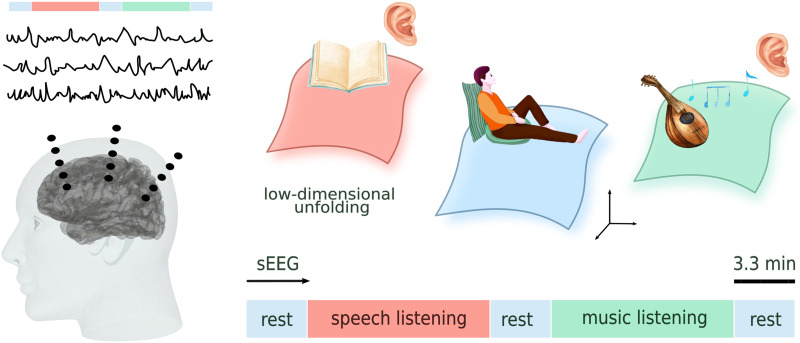
Brain activity was recorded using sEEG. Participants passively listened to a 10-min storytelling segment and a 10-min music piece, both embedded within three resting-state sessions (each lasting 3.3 min). Latent system evolution during the different tasks is captured by a low-dimensional unfolding of the neural space. Comparing dynamical changes across neural manifolds enables a comparative analysis of complexity.

To address these points, we leveraged a dataset in which the brain activity of 19 epileptic patients was recorded using stereotactic electroencephalography (sEEG) during naturalistic speech and music listening (Figure 1). The ecologically relevant stimuli provide a genuine perspective on speech and music listening complexity in a naturalistic scenario. Furthermore, the sEEG signal provides anatomically precise information about the functionally selective engagement of neuronal populations at the millimeter scale and the temporal dynamics of their engagement at the millisecond scale, which are necessary for depicting accurately the neurophysiological underpinning of specific cognitive processes (Mercier et al., 2017; Preti, Bolton, & Van De Ville, 2017). We designed a novel methodology aimed at mapping the dynamical changes underlying the system’s evolution in a neural manifold across diverse tasks, enabling quantitative and comparative analyses of complexity. This method accounts for probabilistic density evolution within low-dimensional spaces defined by latent variables, offering complementarity to existing techniques for assessing the topological properties and task-related segmentation of the manifolds onto which high-dimensional neuronal activity collapses (Areshenkoff et al., 2022; Busch et al., 2023; Chaudhuri et al., 2011; Fan et al., 2016; Kato et al., 2015; Kuo, Chen, & Chen, 2018; Low, Lewallen, Aronov, Nevers, & Tank, 2018; Nieh et al., 2021; Rackauckas & Nie, 2017; Rubin et al., 2019; Rué-Queralt et al., 2021; Vidal et al., 2012).

We hypothesize that speech and music listening display more complex large-scale dynamics, as compared with rest. The hypothesis is based on the more complex cognitive demands that music and speech listening impose on the brain. To further clarify the interpretation of our findings, we first investigate a simple in silico model with known dynamics.

## RESULTS

**Figure 2.**
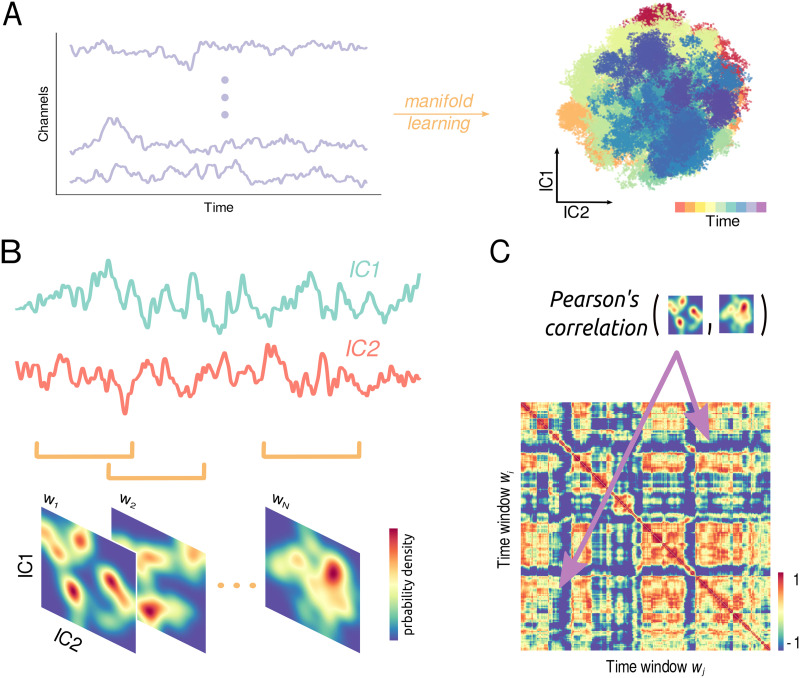
Schematic picture of the construction of the MDF matrix. (A) Through manifold learning techniques, signals extracted from high-dimensional datasets are projected onto a 2D space in which the underlying dynamics of the system takes place. (B) Time series of the two obtained components are segmented into overlapping time windows using a sliding window approach. For each time window, a probability density map is estimated from the distribution of the components in the previously obtained 2D space. Despite the complex manifold architecture spanned by the independent components, simpler patterns emerge in the probability distribution flow, corresponding to the transitions between different system states. (C) Computing the Pearson’s correlation between each pair of these probability densities yields the MDF matrix. Each entry *ij* quantifies the degree of similarity between the probability density map at time window *w*_*i*_ and that at time window *w*_*j*_. The MDF captures the similarity in exploration of the latent space between different segments and allows for the extraction of quantifiable metrics of the system’s inner dynamics complexity.

For simulated or empirical data, we performed an independent component analysis (ICA) on the time series and we projected their time evolution on two components (Figure 2A). Then, the density maps of the system’s trajectory in different time windows (see Figure 2B and the Materials and Methods section) were correlated between one another to obtain the Manifold Density Flow (MDF) matrix (Figure 2C). In this way, the element MDF(*t*_1_, *t*_2_) corresponds to the correlation between the density maps of the system trajectory in the space of the two components of the ICA, during the time window *w*_1_ (starting at instant of time *t*_1_) and the time window *w*_2_. In other words, MDF(*t*_1_, *t*_2_) measures the extent to which the exploration of the latent space in *w*_1_ and *w*_2_ is similar. Considering all the time windows allows us to quantify the complexity of the system’s evolution, in terms of the thoroughness of the exploration of the manifold onto which it is embedded. Estimating the extent to which the manifold is (un)stable across time is a direct measure of its complexity. Intuitively, more complex dynamics, characterized by a greater number of attractive states, evolve more smoothly onto a low-dimensional space, making large jumps less frequent; this behavior is reflected in lower variance of the MDF variance. This information about the evolution of the dynamics could not be unambiguously captured by the mean of the MDF, since different types of dynamics might produce the same mean MDF. Note that, given the symmetric nature of the MDF matrix, that is, MDF(*t*_1_, *t*_2_) = MDF(*t*_2_, *t*_1_), we optimize our calculations by focusing only the upper triangular part of the matrix.

### Simulations of Known Dynamics

**Figure 3.**
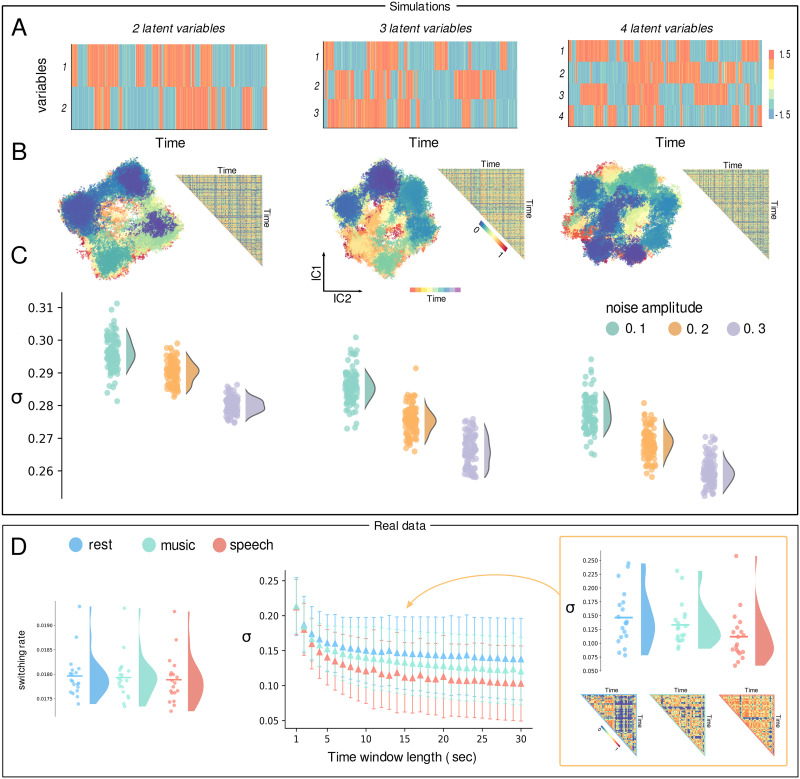
Top panels: Simulations. From left to right the simulated systems with an increasing number of state variables (2, 3, and 4). (A) Separated time series, represented in a carpet plot, of the bistable variables. (B) Representation of the time series in the space of the two components of the ICA, alongside with the upper triangular part of the MDF. (C) Distributions, extracted from 100 samples (100 simulations performed per each condition), of the standard deviations σ of the MDF matrices. The colors indicate varying levels of noise, which correlates with the switching rate, ranging from low (green) to high (purple). Variance in the MDF matrix decreases either when increasing in the number of variables or the amplitude of noise, mirroring the increase in the number of states arising from the superposition of bistable regimes and the switching between them. Bottom panels: Real data. (D) On the left are the distributions of the switching rates, computed averaging the number of switches after having binarized the data (threshold = 2.5 standard deviations); no significant differences are observed between speech, music, and rest conditions (Wilcoxon test: *p* > 0.05). At the center, the means of the distributions of the standard deviation *σ* of the MDF matrices plotted against different numbers of time window length (in seconds) employed in the computation of the MDF. Speech and music listening display higher complexity than rest (CBPT: *p* = 0.005 and *p* = 0.026, respectively), while music listening maintains simpler dynamics compared with speech (*p* = 0.033). This behavior is more clearly illustrated on the right, where we present the distributions for a 15-s window length used to compute the MDF, along with the upper triangular sections of the MDFs for one participant across the three different tasks.

We first utilized a theoretical model with known dynamical complexity to test this interpretation of our method. We simulated a dynamical system composed of *N* = 2, 3, 4 independent variables (Figure 3A). The time evolution of each variable was produced by a cubic differential equation (see the Materials and Methods section). Noise allows for intermittent transition between down- and up-states, corresponding to two equilibrium points, with more noise resulting in more frequent transitions. Note that the number of attractive states scales with the number of variables, as observed from the projection of the system into the two ICA components (Figure 3B). A total of nine scenarios were simulated by manipulating the number of variables and the amount of noise, the latter directly affecting the switching rate between the different states of the system. With the simulated dynamics, we computed the MDF matrix using overlapping windows with a length of 800 time points and a slide of 10 time points. We observed a significantly lower standard deviation in the MDF either when increasing the amount of noise, that is, the switching rate, or the number of independent variables, that is, the number of states explored by the system (Figure 3C).

### During Speech and Music Listening Brain Activity Explores Richer Dynamical Patterns

Our results on synthetic data suggest that a decreasing standard deviation of the MDF could be due either to an increase in the number of states explored by brain dynamics or to a higher noise-induced switching rate. To assess whether there is a significant difference in the switching rate, we binarized the data using different thresholds (2–3 standard deviations; step = 0.1). Then, we counted the number of “switches” for each brain region, that is, how often the region crossed the threshold, and averaged across regions for each participant and condition. For all the thresholds tested, we did not find any significant difference between speech, music, and rest (Wilcoxon test: all *p* > 0.05) (Figure 3D, left). Following this, we computed the MDF matrix during the three conditions using overlapping windows with lengths varying from 1 to 30 s and a slide of 100 ms. The standard deviation of the MDF during both speech and music listening is significantly lower than during rest (cluster-based permutation test [CBPT]: *p* = 0.005 and *p* = 0.026, respectively; Figure 3D, center and right), with the standard deviation of the MDF during music listening being significantly lower than during speech (*p* = 0.033). This supports the hypothesis that brain activity explores more states during speech than music listening, and fewer during rest. This interpretation suggests that complex cognitive functions need to be sustained by correspondingly more intricate brain dynamics. Importantly, our results are not driven by the complexity observed in the auditory channels. Repeating the analysis on channels implanted outside the Heschl’s gyrus (Supporting Information S4) shows a similar trend, confirming that the observed differences in brain dynamics are likely due to higher cognitive processes. Furthermore, to assess the efficacy of our methodology, we conducted a comparative analysis between our results and those derived from the use of the standard deviation of the dynamic functional connectivity (dFC) matrix (Preti et al., 2017) as a metric to quantify and compare complexity across the three distinct conditions. We show how the use of this metric fails to tell the three conditions apart (CBPT: *p* > 0.07) (Supporting Information S1). Notably, our findings are confirmed when using three independent components instead of two, as detailed in the Supporting Information S2.

### Brain Dynamics Is More Similar Across Subjects During Speech Listening

**Figure 4.**
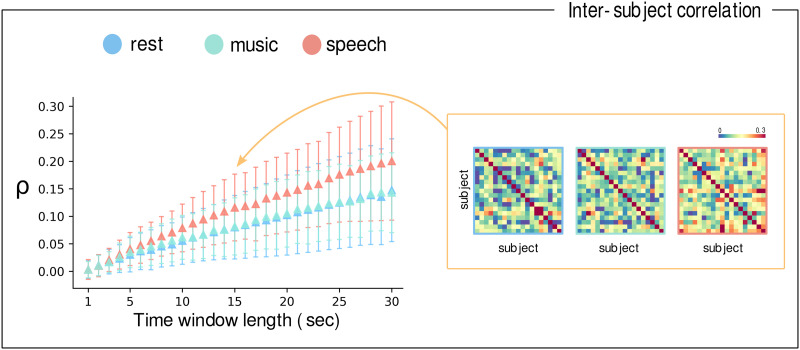
Synchrony in brain dynamical patterns. On the left, the mean correlation matrices (*ρ*) between the MDF matrices are plotted against various time window lengths (in seconds) used in the MDF computation. Speech listening exhibits higher synchrony in brain activity compared with music listening and rest (CBPT: *p* = 0.0001 for both comparisons). On the right, the intersubject correlation matrices for speech listening, music listening, and rest are shown, calculated using a 15-s time window for the MDF computation; a more uniform pattern of synchronization could be observed during speech listening.

We conducted an intersubject correlation analysis to evaluate the consistency of brain activity patterns across participants for the three different tasks. Specifically, we assessed Pearson’s correlation between the MDF matrices computed for each subject. We observed significantly higher intersubject correlations during speech listening compared with both music listening (CBPT: *p* = 0.0001) and resting state (CBPT: *p* = 0.0001) (Figure 4).

## DISCUSSION

In this paper, we set out to test the hypothesis that passive speech and music listening demands a more complex interplay among brain regions, as compared with resting state. Such interactions are mirrored in intricate patterns of interdependencies among recorded brain signals. To quantify these, we analyzed a dataset collected using stereotactic electroencephalography (sEEG) to measure brain activity of 19 epileptic patients during naturalistic speech and music listening and during resting state. According to our hypothesis, we found a growing richness of the dynamical repertoire from rest to music to speech, as quantified by the corresponding diminishing standard deviation of the MDF, which expresses the ability to occupy particular dynamical states and transition between them. This is in accordance with previous literature showing that the presence of tasks pushes the brain dynamics toward configurations that would be otherwise inaccessible during resting state (Areshenkoff et al., 2022).

Our results can be framed within the literature that uses dimensionality reduction techniques to distinguish the dynamics that are induced by different natural stimuli (Areshenkoff et al., 2022; Gonzalez-Castillo et al., 2023). However, somewhat differently than with fMRI, dimensionality reduction is notoriously challenging when dealing with neurophysiological data. Classical manifold approaches focused on the classification of the states constituting the latent space, while disregarding the information about the flow among such states (Gardner et al., 2022; Ji et al., 2015; Martin et al., 2019; Mercier et al., 2022; Nguyen et al., 2018). To overcome these challenges, our paper proposed an approach to characterize the evolution of the density maps obtained from dimensionality reduction over time, successfully capturing the different dynamical complexity in response to different naturalistic stimuli. Our approach is influenced by choices such as the time window duration. We show that our results are generally stable over different windows’ durations, and we highlight that these choices allow us to characterize and investigate different temporal scales.

Of note, we measure the complexity of the dynamics in terms of the standard deviation of the MDF. This metric captures changes in the system’s dynamic patterns, reflected by shifts in trajectories and transitions between attractors in the phase space. Specifically, it could serve as a quantitative and comparative measure of the speed of transitions between brain dynamical states, accounting for the diversity of states (or attractors in the neural manifold) and the likelihood of transitioning from one to another. In our toy model, a state is defined as the combination of active and nonactive units, and we show in a synthetic model that a higher number of possible visited states corresponds to a lower standard deviation of the MDF. When the number of states is very low, the MDF will be more uniform, resulting in a very low standard deviation. As the number of states increases, the standard deviation initially rises due to more distinct attractors, but then decreases when the states become more numerous and closer in phase space. Transitions between them become shorter and smoother, leading to a more uniform MDF and lower standard deviation. However, one challenge is that higher noise might artificially increase the complexity of state space exploration, simply by increasing the probability of any region changing state and so the switching rate. Hence, we show that, keeping the noise level constant, increasing the number of “true” dimensions of the system, that is, its complexity, unambiguously results in lowering the standard deviation of the MDF. In analogy, we have checked that the switching rate of the channels in the real data (i.e., the average number of switches across regions) does not change among conditions, supporting a qualitative change in the dynamics. The comparison of the MDF with dFC demonstrates that the standard deviation of the MDF is particularly well-suited for analyzing complex dynamics within regions exhibiting high levels of integration. However, other features beyond the standard deviation might also be valuable for characterizing the structure of MDF matrices. Future studies should focus on optimizing these descriptions to gain a deeper understanding of the MDF structure. Additionally, our results are robust to the choice of ICA components, as similar trends were observed using three independent components instead of two (see Supporting Information S2).

Our findings suggest that the differences in brain dynamics observed during speech and music listening, as compared with rest, are not merely driven by auditory processing or the complexity of the stimulus itself. In fact, our analysis of channels implanted outside Heschl’s gyrus (Supporting Information S4) reveals that this trend is also present in nonauditory regions. This may indicate that the processing of both speech and music involves widespread brain networks that extend beyond the auditory cortex, reflecting cognitive engagement that is not limited to sensory regions.

We performed an intersubject correlation analysis to evaluate the similarity of brain dynamics across subjects with respect to three cognitive tasks. We observed that brain dynamics during speech listening is more similar than during music listening or rest, indicating a common spatiotemporal structure imposed by the external stimulus. This finding is consistent across various time window lengths used to compute the MDF matrix and further underscore the utility of the MDF in capturing common neural responses among participants, particularly in the presence of a common external stimulus.

In conclusion, in this paper, we used intracranial data recorded during naturalistic speech and music listening and advanced dimensionality reduction techniques to study how the evolution of the interactions among brain regions is modulated by the cognitive conditions. We demonstrated the growing complexity of the dynamics from rest to music to speech. These may reflect the complexity of the neural mechanisms sustaining information integration and segregation during these different cognitive conditions. Future analysis may include a more detailed characterization of the regions involved in these processes.

## MATERIALS AND METHODS

### The Model

The system in the Results section (a) is described by the node equation

$$\dot{x_{i}}=\left(1-x_{i}^{2}\right)x_{i}$$ (1)

in which the nodes are allowed to have intrinsic bistable dynamics. Integration is performed through the stochastic differential equation (SDE) scheme

$$\psi \left(t\right)=f\left(\psi \left(t\right)\right)dt+g\left(\psi \left(t\right)\right)⊗dW\left(t\right),$$

in which *ψ*, *f*, *g*, and *W* are vectors of same dimension and ⊗ indicates the component-wise product. In particular, *ψ* is the state vector *ψ* = [*ψ*_1_, .., *ψ_N_*], with *ψ_i_* =*x*_*i*_, *f* is the vector whose components are derived from the function on the right-hand side of Equation 1, that is, $f_{i}=\dot{x_{i}}$, *g* = [*g*_1_, .., *g*_*N*_], and *dW*(*t*) is a differential of a Wiener process with Gaussian increment. All the nodes are set to have the same level of Gaussian noise $g_{i}=\sqrt{2}\sigma$, where *σ* tunes the noise amplitude. The system is simulated using the just-in-time compilation for SDEs (Ansmann, 2018), a Python implementation of the adaptive integration method proposed by Rackauckas and Nie (2017), with a sampling rate of *dt* = 0.01 over the interval [0, 2000], resulting in a time series composed of 200,000 time points.

### The Manifold Density Flow Matrix

We make use of ICA to embed the continuous time series data into a two-dimensional (2D) space. Subsequently, we segment the resulting time series of the two independent components into smaller time windows of length *L*, employing a sliding window approach where slides occur every *s* time points. Within each time window *T*_*i*_, we generate a smoothed 2D probability density estimation, PDE(*T*_*i*_), using the values of first and second independent components extracted from the time points spanning the interval starting at *T*_*i*_ and ending at *T*_*i*_ + *L*. This involves constructing a 2D histogram of the data using a specified number of bins along both dimensions, capturing the distribution of the data points within the time window interval. For each time window, the probability density is estimated using the boundaries defined by the space spanned by the two independent components across all time points. To achieve a smoother estimate of the probability density, we apply a Gaussian filter with a specified standard deviation to the generated histogram. This filtering process reduces noise in the density estimate, resulting in a smoother representation of the underlying distribution. In our analysis, we employ 128 bins for the 2D histogram and a standard deviation = 8 for the Gaussian filter. Subsequently, we calculate the Pearson’s correlation between each pair of these probability densities (heatmaps) to characterize dynamic changes in the density of the manifold generated by the independent components over time. This correlation analysis yields to a matrix, the MDF, which encapsulates the evolving relationships between the probability density distributions on the low-dimensional space across different time windows. The procedure we propose can be extended, in principle, to an arbitrary number of components (*N*). In such cases, the correlation should be computed between N-dimensional density maps to generate the MDF matrix. In Supporting Information S2, we demonstrate how this can be done using three independent components as an example.

### Intersubject Correlation Analysis

MDF matrices were computed for each participant under the three different cognitive conditions and across various time window lengths. To facilitate comparison, the MDF matrices were flattened into vectors. Pearson correlation coefficients were then calculated between the flattened MDF vectors for different participants, resulting in a correlation matrix that quantifies the similarity in brain response to the cognitive stimulus across subjects.

### Participants

The study involved 19 participants, comprising 10 females, with a mean age of 30 years (range: 8–54 years), all diagnosed with pharmacoresistant epilepsy. All participants were native French speakers. Before undergoing sEEG recordings, neuropsychological assessments were conducted to ensure intact language functions and normal hearing, as per the criteria. Notably, none of the participants had their auditory areas identified as part of the epileptogenic zone, confirmed by an experienced epileptologist’s assessment. The recordings were conducted at Hôpital de la Timone in Marseille, France. Prior to the experimental session, participants provided informed consent, and the experimental protocol received approval from the Institutional Review Board of the French Institute of Health (IRB00003888).

### Data Acquisition

The sEEG signal was captured utilizing depth electrode shafts with a diameter of 0.8 mm, containing 10 to 15 electrode contacts manufactured by Dixi Medical or Alcis from Besançon, France. Each electrode contact was 2 mm long and was spaced 1.5 mm apart from each other along the shaft. Electrode implantation locations were determined solely based on clinical considerations. Participants were included if their electrode map partially covered Heschl’s gyrus, either on the left or right side. Please note that one electrode records in the Heschl’s gyrus, while the others are spread over the cortex; in Supporting Information S3, we report the implantation map. The cohort comprised 13 unilateral implantations (10 left, 3 right) and 6 bilateral implantations, totaling 271 electrodes and 3,371 contacts. During recordings, patients were situated either in an insulated Faraday cage or in a bedroom setting. In the Faraday cage, participants were comfortably seated in a chair within a sound-attenuated room. Data acquisition was performed using a 256-channel amplifier manufactured by Brain Products, with a sampling rate of 1 kHz and a high-pass filter set at 0.016 Hz. Alternatively, in the bedroom setup, data were collected using a 256-channel Natus amplifier from the Deltamed System, sampled at 512 Hz, and high-pass filtered at 0.16 Hz.

### Experimental Design

Participants in the study passively listened to a 10-min storytelling segment (576.7 s, “La sorcière de la rue Mouffetard”; Gripari P., 2004) and a 10-min music piece (580.36 s, “Reflejos del Sur”; Oneness, 2006), both embedded within three resting-state sessions (each lasting 3.3 min). The order of the speech and music conditions was counterbalanced across participants. For analysis purposes, the three resting-state sessions were concatenated into a single 10-min rest condition. During the experiment, sound presentation was facilitated by different setups depending on the location. In the Faraday cage, a Sound Blaster X-Fi Xtreme Audio, a Yamaha P2040 amplifier, and Yamaha loudspeakers (NS-10M) were utilized. Conversely, in the bedroom setting, stimuli were presented using a Sennheiser HD 25 headphone set. Sound stimuli were presented with a 44.1-kHz sample rate and 16-bit resolution, with speech and music excerpts delivered at approximately 75 dBA.

### Preprocessing

Offline, contact data underwent conversion to virtual channels utilizing a bipolar montage approach, specifically employing a closest-neighbor contact reference. This methodology was chosen to enhance spatial resolution and mitigate passive volume diffusion from neighboring areas (Mercier et al., 2017). Precise localization of the channels was achieved through a procedure akin to the one utilized in the iELVis toolbox (Groppe et al., 2017). Additionally, anatomical localization of the channels was labeled using the Brainnetome Atlas (Fan et al., 2016). Subsequently, bipolar channels located outside the brain were excluded from the dataset, constituting approximately 3% of the total channels. The remaining data underwent band-pass filtering between 0.1 and 250 Hz. If required, a notch filter was also applied at 50 Hz and harmonics up to 200 Hz to eliminate power line artifacts. Finally, the data were downsampled to a sampling rate of 500 Hz.

### High-Frequency Activity (HFa)

The amplitude of high-frequency activity (HFa; 80–120 Hz) was derived by computing the analytic amplitude of four 10-Hz-wide subbands spanning the 80–120 Hz range, using the Hilbert transform technique. Each subband underwent standardization by dividing it by its mean value, and subsequently, all subbands were averaged together (Ossandón et al., 2012; Vidal et al., 2012). In order to optimize computational efficiency, the data were downsampled to a frequency of 100 Hz.

### Artifact Rejection

Channels exhibiting a variance exceeding two times the interquartile range, a nonparametric estimate of the standard deviation, were identified and labeled as artifacted channels. Subsequently, for the remaining channels, time segments displaying activity surpassing five standard deviations were flagged as artifacted and replaced with the mean of the signal. This approach ensured preservation of the temporal structure of the data, which is essential for evaluating intersubject correlations.

### Statistics

To identify significant group differences between speech and music listening and resting-state conditions, we employed a CBPT (Maris & Oostenveld, 2007) to the results obtained using the MDF (Results section) and dFC (Supporting Information section) metrics. In particular, we performed the test on the differences between paired conditions. For the results obtained using the MDF metric, only vertices with data values exceeding a cluster-forming threshold of 1 were used to form clusters. For the dFC metric, a range of cluster-forming thresholds from 0.5 to 3.0, with a step size of 1, were tested, confirming that no significant differences were found across these varying levels. In each case, we used 10,000 permutations. Furthermore, to evaluate potential differences in switching rates among the three conditions (Figure 3D, left), we conducted a Wilcoxon test between the paired distributions corresponding to the three tasks.

## ACKNOWLEDGMENTS

This work was co-funded by the European Union (ERC, SPEEDY, ERC-CoG-101043344).

## SUPPORTING INFORMATION

Supporting information for this article is available at https://doi.org/10.1162/netn_a_00422.

## AUTHOR CONTRIBUTIONS

Claudio Runfola: Conceptualization; Formal analysis; Investigation; Methodology; Software; Visualization; Writing – original draft. Matteo Neri: Conceptualization; Data curation; Investigation; Writing – original draft; Writing – review & editing. Daniele Schön: Investigation; Resources; Supervision; Validation; Writing – original draft; Writing – review & editing. Benjamin Morillon: Data curation; Funding acquisition; Investigation; Resources; Supervision; Validation; Writing – original draft; Writing – review & editing. Giovanni Rabuffo: Conceptualization; Investigation; Methodology; Software; Validation. Pierpaolo Sorrentino: Conceptualization; Investigation; Methodology; Supervision; Validation; Writing – original draft; Writing – review & editing. Viktor Jirsa: Funding acquisition; Investigation; Project administration; Supervision; Validation. Agnès Trébuchon: Resources.

## FUNDING INFORMATION

Viktor Jirsa, HORIZON EUROPE Innovative Europe (https://dx.doi.org/10.13039/100019186), Award ID: SGA 101147319.

## Supplementary Material



## TECHNICAL TERMS

Brain dynamics: The study of the temporal changes in brain activity patterns, essential for understanding cognitive processes and neural function.
Stereotactic electroencephalography (sEEG): A technique for recording electrical activity in the brain using implanted electrodes, providing high spatial and temporal resolution.
Neural manifold: A low-dimensional space representing the relationships and patterns of brain activity across different cognitive states.
Independent component analysis (ICA): A statistical method for separating mixed signals into their original independent sources, often used in neuroimaging studies.
Manifold Density Flow (MDF): A matrix representation that captures how the distribution of brain activity changes over time in a simplified space.
Cluster-based permutation test: A statistical method used to identify significant differences between conditions by examining clusters of data points.
Dynamic functional connectivity: A measure of the changing functional relationships between different brain regions over time, reflecting dynamic brain network activity.
Stochastic differential equation (SDE): A mathematical equation used to describe systems influenced by random noise, commonly applied in modeling brain signals.
Probability density estimation (PDE): A technique for estimating the likelihood of a variable falling within a certain range, useful in analyzing data distributions.
High-frequency activity (HFa): Brain signal oscillations in the 80–120 Hz range, often linked to cognitive processes and sensory perception.
