# Supplementary material for: Complexity in speech and music listening via neural manifold flows
Source: Netw Neurosci. 2025 Mar 5;9(1):146–58. doi: 10.1162/netn_a_00422 (PMC11949541; doi:10.1162/netn_a_00422)
Supplement: Supplementary file 1 [file netn-9-1-146-s001.pdf]

Supplementary Material

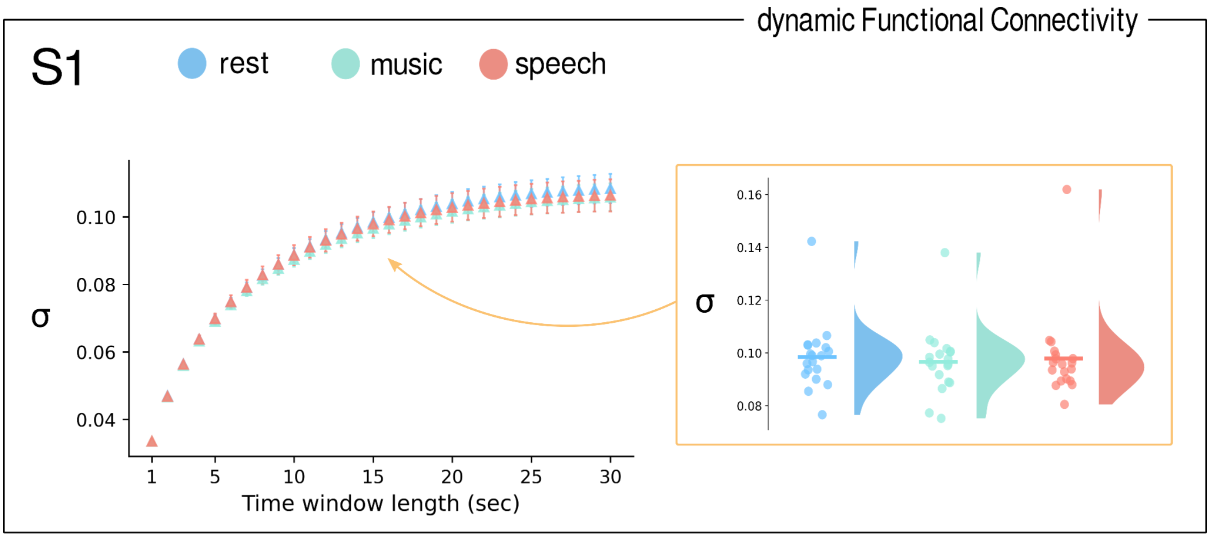

Results using dynamic Functional Connectivity as a metric. On the left, the means of the distributions of the standard deviation σ of the dynamic Functional Connectivity (dFC) matrices plotted against different numbers of time window length (in seconds) employed in the computation of the dFC. No significant differences are observed between speech, music, and rest conditions (P>0.07). This supports the effectiveness of our methodology.

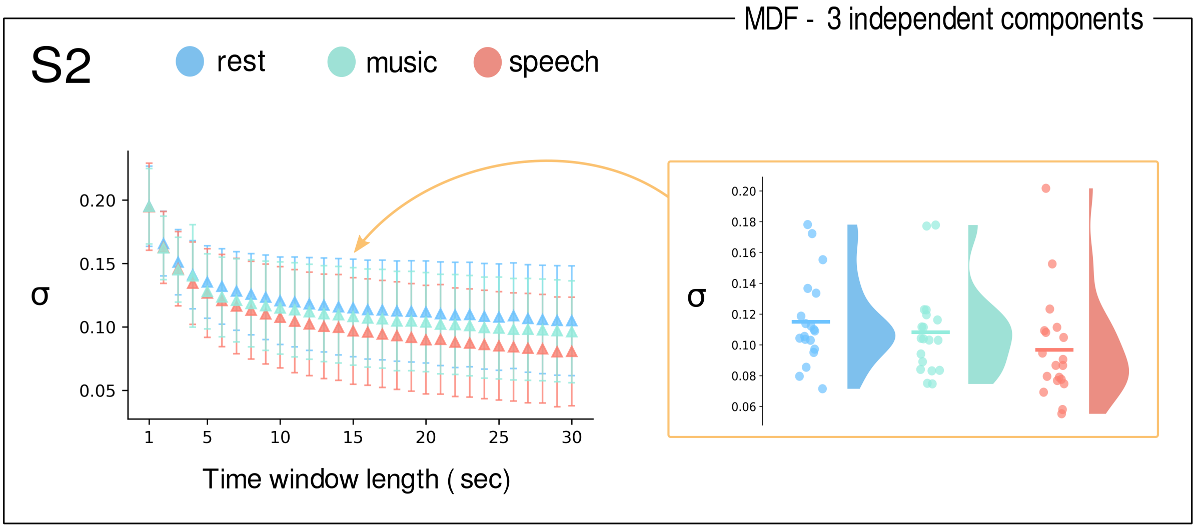

Results using three independent components to build the MDF. On the left, the means of the distributions of the standard deviation σ of the MDF matrices plotted against different numbers of time window length (in seconds) employed in the computation of the MDF. Again, speech and music listening display higher complexity than rest, while music listening maintains simpler dynamics compared to speech. This confirms the trend observed when using only two independent components in the construction of the MDF. On the right, the same distributions corresponding to the case in which the window length used to compute the MDF is set to 15 seconds.

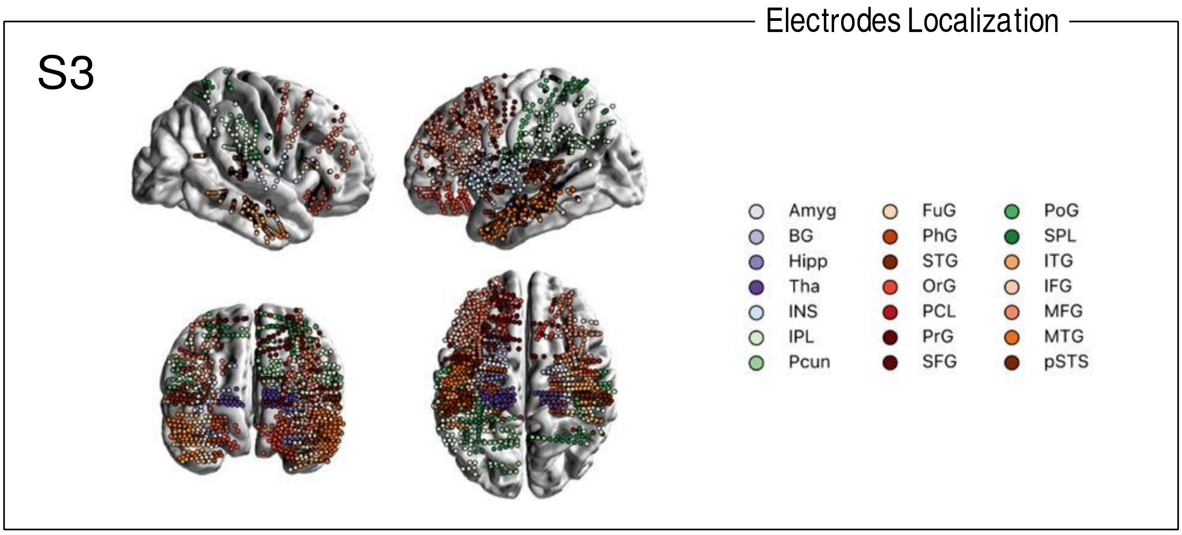

sEEG Electrodes Localization Across Anatomical Regions. Abbreviations are based on the Brainnetome Atlas (Fan et al., 2016). Figure adapted from te Rietmolen et al. (2024).

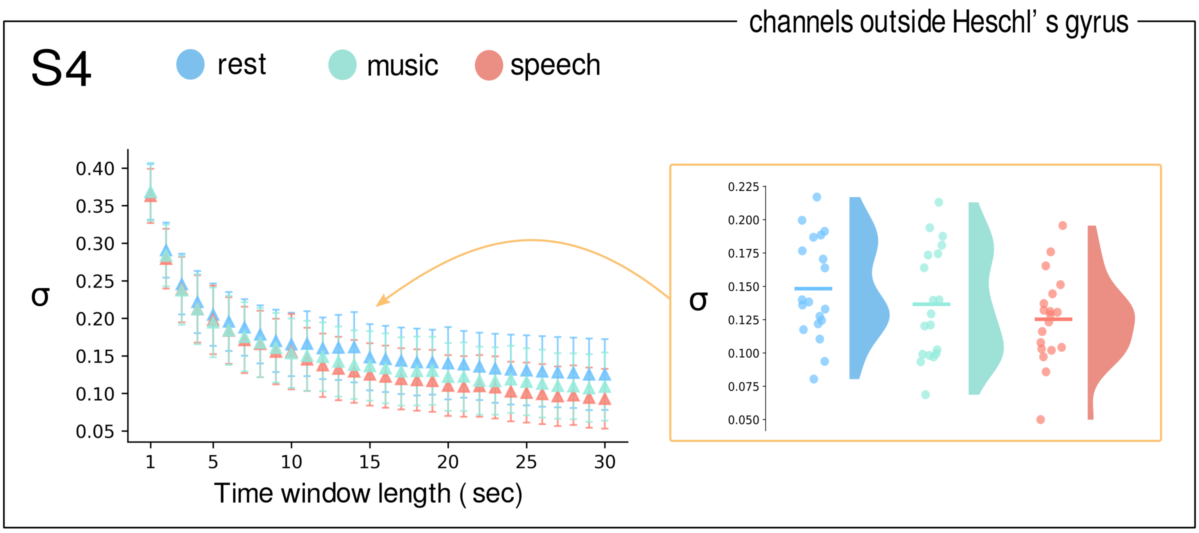

Results performing the MDF on the channels implanted outside the auditory cortex. On the left, the means of the distributions of the standard deviation σ of the MDF matrices plotted against different numbers of time window length (in seconds) employed in the computation of the MDF. Again, speech and music listening display higher complexity than rest, while music listening maintains simpler dynamics compared to speech. This confirms the trend observed when the MDF is performed on all the channels. On the right, the same distributions corresponding to the case in which the window length used to compute the MDF is set to 15 seconds.
